# Nanopharmaceutics: Part I—Clinical Trials Legislation and Good Manufacturing Practices (GMP) of Nanotherapeutics in the EU

**DOI:** 10.3390/pharmaceutics12020146

**Published:** 2020-02-11

**Authors:** Eliana B. Souto, Gabriela F. Silva, João Dias-Ferreira, Aleksandra Zielinska, Fátima Ventura, Alessandra Durazzo, Massimo Lucarini, Ettore Novellino, Antonello Santini

**Affiliations:** 1 Department of Pharmaceutical Technology, Faculty of Pharmacy (FFUC), University of Coimbra, Pólo das Ciências da Saúde, Azinhaga de Santa Comba, 3000-548 Coimbra, Portugal; gabriela.mgfs@gmail.com (G.F.S.); j.dias.ferreira@outlook.pt (J.D.-F.); zielinska-aleksandra@wp.pl (A.Z.); 2 CEB - Centre of Biological Engineering, University of Minho, Campus de Gualtar 4710-057 Braga, Portugal; 3 Department of Biochemistry and Human Biology, Faculty of Pharmacy, University of Lisbon, 1649-003 Lisbon, Portugal; fatima.ventura@infarmed.pt; 4 CREA-Research Centre for Food and Nutrition, Via Ardeatina 546, 00178 Rome, Italy; alessandra.durazzo@crea.gov.it (A.D.); massimo.lucarini@crea.gov.it (M.L.); 5 Department of Pharmacy, University of Napoli Federico II, Via D. Montesano 49, 80131 Napoli, Italy; ettore.novellino@unina.it

**Keywords:** nanopharmaceutics, legislation, clinical trials, quality, safety, GMP

## Abstract

The latest advances in pharmaceutical technology are leading to the development of cutting edged approaches to produce what is now known as the “Holy Grail” of medicine—nanopharmaceutics. Over the latest decade, the pharmaceutical industry has made important contributions to the scale up of these new products. To ensure their quality, efficacy, and safety for human use, clinical trials are mandatory. Yet, regulation regarding nanopharmaceuticals is still limited with a set of guidelines being recently released with respect to compliance with quality and safety. For the coming years, updates on regulatory issues about nanopharmaceuticals and their use in clinical settings are expected. The use of nanopharmaceuticals in clinical trials depends on the approval of the production methods and assurance of the quality of the final product by implementation and verification of the good manufacturing practices (GMP). This review addresses the available legislation on nanopharmaceuticals within the European Union (EU), the GMP that should be followed for their production, and the current challenges encountered in clinical trials of these new formulations. The singular properties of nanopharmaceuticals over their bulk counterparts are associated with their size, matrix composition, and surface properties. To understand their relevance, four main clinical trial guidelines, namely, for intravenous iron-based nanopharmaceuticals, liposomal-based nanopharmaceuticals, block copolymer micelle-based nanopharmaceuticals, and related to surface coating requirements, are described here.

## 1. Introduction

The past decades have been successful in developing several nanopharmaceutical formulations for the treatment of cancer, infectious diseases, neurodegenerative diseases, diabetes, etc. [1,2,3,4,5,6]. Per definition, a nanopharmaceutical should have at least one dimension in the nanoscale—from 1 nanometer to 100 nanometers—for which the properties considerably differ in comparison to the bulk counterpart [7]. Nanopharmaceutics stand for pharmaceutical products at the nanoscale, developed with the aim to improve the physicochemical, biopharmaceutical, and pharmacokinetic properties not only of already existing drugs, but also of new molecular entities with biological activity. Nanopharmaceuticals comprise the loading of active pharmaceutical ingredients (APIs) within a nanocarrier with the purpose to improve their solubility, extend their half-life, improve pharmacokinetic properties, obtain a modified release profile, reduce acute/chronic toxicity, or even achieve site-specific targeting [4,8,9,10,11]. The use of nanocarriers to load APIs may offer the opportunity to reduce the dose required for a therapeutic response, attributed to the site-specific delivery of the API with limited systemic distribution [12,13]. The therapeutic index is also improved as nanopharmaceutics can overcome several anatomic and physiological barriers. Albumin-based nanoparticles, liposomes, PEGylated proteins, and polymeric micelles are just a few examples of a range of nanopharmaceuticals already approved for human use.

From the design of a nanopharmaceutical product to its marketing, regulatory guidelines are used to ensure efficacy, quality, and safety of the final product [14]. One of the most challenging steps is the scaling-up from the small laboratory batch to large industrial volumes of formulations [15]. The optimized physicochemical, pharmacokinetic, and biopharmaceutical properties of the small laboratory batch should be kept along the scale-up process to ensure both the in vitro and in vivo performances of the nanopharmaceutical, thereby limiting the risk of toxicological events. good manufacturing practices (GMP) are thus instrumental to ensure the quality of processes and obtained products. The European Technology Platform on Nanomedicine (ETPN) projected as a Horizon 2020 aim the following purposes: “GMP manufacturing pilot lines for clinical batches, which will both assist academic groups and, especially, SMEs (Subject-Matter Expert) to develop their nanomedical materials for validation in clinical trials, before transfer to CMOs (contract manufacturing organizations); a strong link with existing European clinical networks or organizations to help transfer and provide efficient early clinical trials in nanomedicine.”

Yet, regulations are still immature with respect to nanopharmaceuticals while the European Union (EU) allows a diversity of adaptations of the main guidelines at the national levels to comply with the specificities of each Member State [16]. Regardless of the national specifications, in vitro and in vivo studies must be accomplished together with compulsory clinical trials. A diversity of shareholders is currently focused on a translational approach to make research and development of nanopharmaceuticals more effective and profitable.

## 2. Regulatory Framework of Clinical Trials

The launch of a new medicine comprises a complex pathway that requires research and development for a period of 5 up to 10 years with a massive parallel investment. Research firstly starts with the understanding of the pathology as a whole and the identification of potential therapeutic targets, followed by the selection of some API, which should be active enough to limit the progression of the disease. During the pre-clinical phase, which may take between 3 and 6 years, the selected API is tested in vivo in animal models in order to collect relevant in vivo data for the clinical trials that come next.

A clinical trial can be defined as the study which compares the effects and values of a given drug formulation with a control process, both developed in humans. The principal feature of a clinical trial is its prospective nature, different from retrospective studies [17,18]. To collect relevant clinical data, the volunteers (patients or healthy individuals) participating in the clinical trial must be continuously tracked over time to attain the best possible description about the effects of the treatment under study. The clinical trial assesses not only the parameters of efficacy and safety of a specific treatment but is also used to determine pharmacokinetics parameters (i.e., absorption, distribution, metabolism, and excretion), to estimate the risk of adverse reactions and to assess the efficacy and safety of a product in comparison to a currently used treatment [19,20,21].

The clinical trials comprise four phases— I, II, III, and IV—the classification being dependent on the stage of development of the employed drug, as well as on the post-marketing authorization, which means that the characterization of these phases cannot be strictly defined. Nonetheless, they can be settled according to their main purposes and clinical development.

Phase I comprises studies of Clinical Pharmacology and Toxicity with a first evaluation about drug safety, pharmacokinetics, and pharmacodynamics, using human volunteers who are typically healthy individuals. Drug dosage suitable for the tested health condition is also determined, administering a continuous increase of an applied dose to avoid severe side effects. This phase comprises several volunteers, a number that may vary between 10 and 100 persons enrolled in the trial for 1.5 years.

Phase II comprises studies of Initial Clinical Investigation for Clinical Effect with the purpose of assaying the safety and efficacy of an employed therapeutic on volunteers with a pathology for which the given medicine is specific. These studies have in their basis the comparison between the placebo and controlled design. In this phase, the number of volunteers may vary from 100 to 200, enrolled for a period of 2 years.

Phase III covers the Full-Scale Evaluation of Treatment on which larger clusters of volunteers are used aiming at comparison between the medicine taken into the clinical trial and a gold-standard in that field of therapeutics. Phase III trials must validate the effectiveness of the treatment under testing, its side effects, and even interactions. This phase is usually run for an extended period, about 3 years. The design of the studies in this phase is double-blinded and randomized attempting an approach to the real situation.

Phase IV is the stage of Post-Marketing Surveillance and aims at monitoring the medicine after marketing approval. Here, the summary of drug product (SDP) is the focus of the research, targeting the continuous monitorization of the side effects of the medicine.

### 2.1. European Legislation

The last decade was particularly prominent on the release of European legislation about clinical trials. Regarding the harmonization of administrative requirements, regulations and laws of different Member States, the Directive 2001/20/EC was amongst the first efforts arising from the European Parliament to ensure Good Practices during the implementation of clinical trials using medicinal products on humans. This process of harmonization was conducted by the International Council on Harmonization (ICH) which introduced very effective and mandatory guidelines, as ICH E6, aiming at good clinical practices (GCP), including EU legislation through the directives 2001/20/EC, 2005/28/EC, and 2003/94/EC. The EU also implemented, through European Medicines Agency (EMA), an assembly of regulations regarding the development of medicinal products in the EU—the EudraLex. European legislation regarding pharmaceuticals is gathered in Volumes 1 and 5, whilst the guidelines and procedures for clinical trials are provided in Volume 10 [22].

The Directive 2001/20/EC describes the both the implementation of GCP and the volunteers used in trials. It also allowed the implementation of deadlines on the process of clinical trials, and the setup of a database, the EudraCT, which fostered some competition within the pharmaceutical industry due to the rigorous and clear research and development process [23].

The directive 2003/94/EC, of 8th of October 2003, describes the main guidelines on GMP regarding the production of medicines and investigational medicinal products (IMPs) for human use, which shall follow what is established in Volume IV of Eudralex. The conformity with the GMP is a mandatory element for all the products, while those which are produced outside the EU must hold a certificate that ensures that GMP are followed. Besides, the up-to-date scientific information to the manufacturing process should be easily reviewed. The development of IMP requires tight legislation due to the employment of the manufactured medicines on human subjects—since these are not yet tested. The quality and efficacy of the developed IMP are ensured through the implementation of GMP [24].

In the Annex number 13 of Volume IV of the Eudralex is the information exclusively related to IMP which is “a pharmaceutical form of an active substance or placebo being tested or used as a reference in a clinical trial, including a product with a marketing authorization when used or assembled (formulated or packaged) in a way different from the authorized form, or when used for an unauthorized indication, or when used to gain further information about the authorized form”. IMP manufacturing requires a higher level of regulation due to the possible hazard arising from the unknown effects of such products in comparison to others that are marketed, as the former are subject of several different clinical trials and, thus, the product must comply to all requirements as packaging, labeling, and analytical control. These points are so critical that a bad clinical trial design may lead to a failure in the production of a new IMP. Quality performance is a first-order feature and must consider the guidelines described in GMP in such a way that the equipment and clinical trial stage shall meet the requirements of IMP. Concerning the required credentials, they must be clearly described, specified, and subject to updates as they become available. The uniformity of each produced batch must be acceptable to ensure the specification of the product. The parameters of manufacturing should also be determined accurately and well-reasoned, keeping a recording of all the values and parameters to further rationalize the marketing authorization application. The process of packaging is critical to prevent accidents. Depending on the type of clinical trial design, other products as non-investigational medicinal product (NIMPS) or rescue medication may be employed in the study. Additionally, as there is a need for a placebo, this latter must have the same outer aspect as the IMP, but apart from the active molecule(s). The process of packaging must therefore be carefully carried out by trained staff.

Directive number 2003/94/EC specifies the conditions of labelling on IMP, which does require exceptional care. The most important characteristics in this process are (i) the name, address, contact number of the sponsor of clinical trial, contract research organization or investigation must be present; (ii) the pharmaceutical form of dosage, the route of administration, the dosage units and, if the clinical trial is open, the identification of substance and its potency shall be described; (iii) a code of identification of contents in the package and a code for the trial must be present; (iv) trial subject identification number or treatment number and visit number (when relevant) must be distinct as well as the identification of the investigator; (v) a label with the designation “For clinical trial use only”, “Keep out of reach of children”, and the period of use of the product must be described. Batch records, production settings, and GMP consent are the required criteria to be fulfilled for a qualified and certified person to be able to release the IMP. If the aim is to test an IMP in a clinical trial, it must comply with the requirements of Annex 13 and, if absent, it must be completely justified [25].

Towards a submission of a clinical trial procedure, a document named Investigational Medicinal Product Dossier (IMPD) is necessary with a format similar to the common technical document (CTD), regarding the Module 3 of Quality. The IMPD may also be subject of simplifications depending on the circumstances.

The detailed guidelines and principles aimed at the employment of GCP for research purposes of IMP for human use are described in the Directive 2005/28/EC, 8th of April 2005. It also elucidates the required supplies to import such products, to commercialize them, or even the documents in respect to the clinical trials and the procedures of inspection. The safety and shielding of the participants in clinical trials is the main topic for which Ethics Committees were developed in each Member State [26].

The International Harmonization Council (ICH) has been set up to address key differences in drug development in the United States, EU, and Japan. The ICH encompasses guidelines for Safety, Quality, and Efficiency issues as well as other topics, including Medical Dictionary for Regulatory Activities (MedDRA) and CTD [27].

In 1996, the ICH established the guideline E6 on GCP. The ICH also stated that those would be conducted in harmony with the ethical ideologies of the Declaration of Helsinki. The described document should contain the entities related to the performed clinical trials and the responsibility that each one has in the process. It additionally defines the type of format that clinical trials documents should possess. The procedure of GCP was not legally derived until the emergent Directive 2001/20/EC which stressed that GCP should compose the legislation of each country on what clinical trials are concerned.

Institutional Assessment Board/Independent Ethics Committee (IRB/IEC) stands as the entity responsible for the assurance of the well-being of participants in clinical trials. An informed consent must be signed by the subjects participating in the trial confirming their clear knowledge and awareness about the aims of the process, the procedures applied, the rights and responsibilities of the participant. The informed consent is designed and reviewed by IRB/IEC. The possible risks of the trial and the purpose must be thoroughly explained to the participants. The IRB/IEC also reviewed other documents as study protocol and adjustments to it, investigator’s brochure (IB), to require, or not, modifications in order to achieve the best design of the trial. The investigator must be a qualified and certified professional with long training in the field of GCP and must hold the responsibility for the trial conduct. His/her curriculum vitae must be at the disposure of the sponsor IRB/IEC as well as to the regulatory affairs authority. The investigator has the obligation to know how to use the IMP always following the IB. The sponsor has as main concern to ensure the quality of the obtained results and to protect the participating subjects over the course of the trail. To reach such purpose, a system based on the quality supervision and risk is employed comprising the identification, the evaluation, the control, the communication, the review, and the reporting. The sponsor always holds the responsibility for ensuring data collection. Nevertheless, his/her functions may be transferred to a contract research organization (CRO).

With respect to the clinical trial protocol, the inclusion of protocol title, the identification number, the starting date, and the name of the investigator must be provided. Additional information includes the concerns and criteria for clinical trial, as well as its design, inclusion, and exclusion criteria, the treatment of subjects, the process of evaluation of effectiveness and safety, and the statistics process. The IB must include data concerning the clinical and non-clinical outcomes about IMP that help to appreciate the specifications on some protocols as the dose and frequency of treatments and the employed methods of administration [28].

Other documents concerning the GCP do also exist and take into account the phases of a developed study—the time before, during, and after—which serves as complement to the information conducted in the study. Furthermore, the regulation number 536/2014 concerning the clinicals trials applied to medicines for human use was removed by the Directive 2001/20/EC [29]. After the implementation of this regulation, the number of clinical trials was reduced due to the time and resources. The formal regulation form has more advantages when compared to a given directive, due to the stability achieved among researchers and sponsors of clinical trials since the application is equally leveled for all the purposes without exceptions. The European Commission defines the current regulation with some topics—it must have a single, updated, and structured platform for submission forms (EU portal); the submission shall be performed with an official arrangement which is settled in the Annex I of the given Regulation; the process must be clear and transparent from the beginning to the end; the clinical trial application must possess a well-defined deadline; the Ethics Committee must be related to the evaluation of the procedure in accordance to the law of the given country but has to respect the defined timelines; the reports must be simplified in order to avoid repeated information and to spare the sponsors; the applications must run through two rounds, first, the Members States equally concerned about it and, secondly, by each Member State individually; the EU controls the way the process is being carried out to ensure the supervision and that the rules are being followed. When the clinical trials are performed outside the EU, they must comply with all the laws in rule within EU [30].

Concerning the Portuguese National Legislation as an example, the Law Number 21/2014 regulates the procedures of clinical research at a national level and compelled the creation of a National gateway for submission of proposals for clinical trials. It also included clinical trials with products of medicinal nature applied to humans, medical devices, and cosmetics. According to the previous law, the electronic submission of all documents required for clinical trials approval, and specific adjustments, may be done. This platform also serves the purpose of allowing the research of data upon studies available. In the following, a National network concerning ethics was also established. These innovations are beneficial for the entire process of submission and approval of a project for clinical trials allowing reduced deadlines and quick approvals arising from the National Authority of Medicines and Health Products (INFARMED) and from the Ethics Committee (CEIC) [31].

The first adjustments implemented on Clinical Investigation Law (CIL) pointed to the clarification of some aspects associated with data accessible for auditors and inspectors about the subjects gathered in a specific study. The implementation of a clinical trial requires, firstly, the assurance that the subjects participating in the trials are protected and that a defined and approved protocol is followed. To ensure that all processes are followed as legally required, the authorities must oversee every document and action related to the clinical trial and verify if the legislation is followed [32]. During the clinical trial an audition is a requirement. The subjects participating in the clinical trials therefore have to sign an authorization for further purposes of accessing data. CIL came into a discussion with National Commission for Data Protection (CNPD) which was based on the blockage of access of the latter to the information of patients. This modification was enforced to make clear the role of the auditors as well as their auditions in the process of clinical trials.

In Portugal, the Law number 21/2014, of 16th April, is an amendment to the Law number 73/2015 and regulates the behavior during clinical trials using medicines for human use. Nevertheless, the European regulation present in the Volume X of Eudralex is also applicable [33]. Yet, due to certain constraints and regarding the creation of a more advantageous regulation in clinical research, the European Regulation number 536/2014 was settled on the 16th of April 2014, concerning the use of medicines for human use in clinical trials. This regulation also brought a higher level of transparency, speed, and homogeneity on what the process of information disposal on clinical trials is concerned.

### 2.2. Nanopharmaceuticals in Clinical Trials

Over the last decades, the number of nanopharmaceuticals being approved for market commercialization expanded intensively and is now a reality in the medical field [34]. Bremer-Hoffman et al. analyzed quite a few platforms and, in 2015, published a document on which it was stressed that between 1996 and that year 131 drugs were included in projects of development directly related with nanomedicines, 69 nanomedicines were related with clinical trials, and 30 nanomedicines were authorized. The results achieved from 2015 until May of 2017 arriving from the EU, and regarding Clinical Trials Register, recorded 18 clinical trials concerning nanopharmaceuticals [35].

The information available online about clinical trials and the literature data demonstrated that nanopharmaceuticals are present in all phases of research and development processes [36]. This type of technology is employed in the therapeutics of a variety of diseases and the category of formulations is also diverse concerning liposomes, nanoemulsions, polymeric micelles, and many others [37,38,39]. The currently exponential use of nanopharmaceuticals is definitively a breakthrough and a need on what the creation of new regulations for the correct development of these products is concerned.

## 3. Good Manufacturing Practices Applied to Nanopharmaceuticals

**Figure 1:**
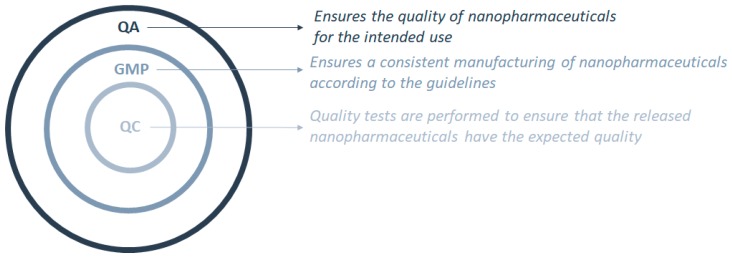
Interconnections between the quality control (QC) process as part of the good manufacturing practices (GMP), which are part of the quality assurance (QA) process.

The exceptional, unique, and singular characteristics of nanopharmaceuticals are associated with their size, matrix composition, and surface properties [4,5,11,40,41,42,43]. As such, the current legislation must suffer a shift to attend the needs of these new types of pharmaceuticals as these are not approachable with the current regulation applied to health products. Further application of these medicines in clinical trials is dependent on the approval of the production methods and the quality assurance of the final product by the implementation and verification of the GMP [39]. GMP are in fact a part of the quality assurance (QA) process that should be implemented to ensure that the nanopharmaceuticals are consistently manufactured following the specifications and with the required quality. Quality control (QC) ensures that the necessary quality check tests are carried out so that the nanopharmaceutical product to be launched has the expected quality for the intended use (Figure 1).

The Food and Drug Administration (FDA) fosters the development of new medicines with added value for human health. Amongst them, some new promises in nanopharmaceuticals are listed [44]. In 2013, FDA launched the Nanotechnology Regulatory Science Research Plan which details the execution and management related with any type of nanotechnologies or nanoproduct derived from those. The aim of such procedure was to mitigate the breach existing in respect to nanopharmaceuticals. There are four principal areas covered by the previously described program: the first is related with the Staff Training and Professional Development; the second is in respect to Laboratory Core Facilities; the third is connected to the Collaborative Opportunities for Research Excellence in Science (CORES) Program; the fourth and last regards the FDA Coordination. With this categorization, an approach to ensure efficacy, quality, and safety of nanotechnologies is aimed.

The existing gap in the legislation of nanopharmaceuticals leads to uncertain decisions when authorizations for marketing introduction of these products are to be approved. To assist pharmaceutical industry to submit the required documentation for the market authorization for a certain medicine, and to clarify certain scientific doubts, European Medicines Agency (EMA) has released the so-called “reflection papers” [45]. Additional regular guidelines are also published by EMA according to the situation under study. In 2010, participants from EU, Australia, Canada, India, Japan, and the US joined the first international scientific workshop on nanomedicines to discuss on the regulatory framework for the development and evaluation of nanopharmaceutics [46]. In July 2019, the EU and the USA finally implemented the mutual recognition agreement (MRA) for inspections of manufacturing sites for human medicines [8], that resulted from the fact that both EU and USA have comparable procedures with respect to GMP inspections for human medicines. Some efforts are also being made in Asia to harmonize the production of nanopharmaceuticals with international regulations [47].

### 3.1. Intravenous Iron-Based Nanopharmaceuticals

The present guideline aims to generate valuable information about non-clinical, pharmacokinetics and quality facts to establish some comparisons with an already-in-market products to attain a market introduction authorization for an iron-based intravenous medicine with nanocolloidal design. The iron-based products are used to treat patients with iron deficits; their structure entails an iron polynuclear core with iron (III)-oxyhydroxide and a carbohydrate on a complex-covering to stabilize the formulation leading to a nanosized structure of aggregates in a colloidal system [48]. According to the current experience, to assure their equivalency, these colloidal systems cannot be analyzed through quality methods only and since the toxicological results are not conclusively defined through pharmacokinetic assays non-clinical information is commonly requested [49]. As a result, a guideline was defined with three main topics: the first, related to quality, shall possess the quality characterization of the product in test and the establishment of a pattern of comparability in terms of pharmaceutical test and reference product; the second, related to non-clinical information, shall contain methods of analysis and biodistribution studies; the third, related to clinical data, shall possess efficacy and safety studies, pharmacokinetic studies, and even pharmacovigilance and a risk management plan.

### 3.2. Liposomal-Based Nanopharmaceuticals

The present guideline aims to create satisfactory information about clinical, non-clinical, and quality facts to set up a marketing introduction authorization for pharmaceutical biosimilar products for intravenous administration with liposomal nature. The loading of an API in a liposome (or bound onto its surface) usually aims for targeted delivery. These products may however show some problems related to their pharmacokinetics and risk of early drug release. To establish the parameters of safety and effectiveness of a novel liposomal product, certain parameters such as stability and pharmacokinetics are required.

Several assays must be applied to the liposomal formulation to be thoroughly tested and to demonstrate its quality and safety as bioequivalence tests are not enough and possible sample deviations occurring in the process of manufacturing may lead to disparities. The present guideline serves the purpose of developing a new liposomal product and comparing it with an already established innovator [50]. To do so, the guideline was defined according to (i) the pharmaceutical quality, which encompasses the establishment of pharmaceutical comparability, the quality characterization, and the pharmaceutical development of the applicant’s product; and (ii) the non-clinical and clinical requirements, which includes the general aspects, the methods of analysis, the non-clinical and clinical studies.

### 3.3. Block Copolymer Micelle-Based Nanopharmaceuticals

This guideline describes the production process of medicinal products based on block-copolymer-micelle structure and the guidance of some of the first stages of clinical trials. The copolymer micelles are engineered through self-assembly of charge-possessing copolymer (with amphiphilic properties in aqueous medium) with the API incorporated into the core of the produced micelle [51]. An index was created considering several steps namely, (i) chemistry, manufacturing, and control: the changes during the development in the manufacturing process, the description, and composition of the product, the manufacturing process as well as the process control, the characterization of quality, the pharmaceutical quality, the product specification, and stability; (ii) general considerations, non-clinical pharmacokinetics and pharmacodynamics, safety, pharmacology, and toxicology; (iii) the considerations for first-in-human studies [52].

### 3.4. Surface Coating Requirements

This guideline highlights some critical points with respect to the generation of medicines with a coat for parenteral administration (e.g., polyethylene glycol (PEGylation) coating). The process of coating improves the time of circulation in plasma by the increase of the hydrophilic character of the surface [53], as well as the stability, being part of the final product. The surface coating influences remarkably the behavior of particles being, therefore, instrumental for defining their efficacy and safety. The description of product must be exhaustive and thorough [54].

## 4. Conclusions

Research on nanopharmaceuticals has increased tremendously over the last two decades, which triggered the publication of guidelines to ensure their quality, safety, and efficacy. The advantages of nanopharmaceuticals come from their morphology, size, and shape which contribute to improving the properties of the bulk counterparts, but they are effective only if the quality is unaltered between batches of manufactured products. A reproducible, scalable production method must be developed and validated. On one hand, good manufacturing practices (GMP) should ensure that guidelines recommended by agencies that control the authorization and licensing of the nanopharmaceutical are followed; on the other hand, clinical trials have to be run to set up the highest benefit/risk ratio.

Further studies should also address applications of nanotechnologies to nutraceuticals [55,56,57,58,59,60,61,62,63], in the perspective of the new branch of “Nanonutraceutical Science” [64,65,66]. Nutraceuticals, which reside in a gray area between pharmaceuticals and food, have been defined recently as “the phytocomplex if they derive from a food of vegetal origin, and as the pool of the secondary metabolites if they derive from a food of animal origin, concentrated and administered in the more suitable pharmaceutical form” [60,62]. It must be mentioned the existing lack of a shared regulatory framework on nutraceuticals including their today inclusion in the food supplement area: e.g., in the European Regulations of 2015 the term nutraceutical is still not recognized notwithstanding the growing impact and interest both in the research area and commercial/market/patents impact, since they bridge the gap from conventional pharmacological therapy with natural substances able to prevent/delay the onset of long term pathological conditions. The nanonutraceuticals formulations to be exploited based on the results obtained with pharmaceuticals, for their efficacy and effect, could be another tool in the arsenal of strategies helpful in managing health conditions, especially in patients who are not eligible for conventional pharmacological therapy [67,68,69,70,71]. Recent examples are the end points reached on the use of milk thistle (*Silybum marianum*) in liver diseases and of the apple phytocomplex against hypercholesterolemy [72,73]. Studies on follow up, use, and compliance of nanonutraceuticals, as shown by recent research in the area [74,75,76], as well as communication strategies and assessment [77], should be carried out.
